# COVID-19 Vaccine-Induced Pneumonitis, Myositis and Myopericarditis

**DOI:** 10.7759/cureus.20979

**Published:** 2022-01-06

**Authors:** Mariya Farooq, Yaser Mohammed, Mansoor Zafar, Dawpadee Dharmasena, Usman Iqbal Rana, Osei Kankam

**Affiliations:** 1 General Internal Medicine, Conquest Hospital, East Sussex Healthcare NHS Trust, St. Leonards-on-Sea, GBR; 2 Internal Medicine, Conquest Hospital, East Sussex Healthcare NHS Trust, St. Leonards-on-Sea, GBR; 3 Gastroenterology and Hepatology, Conquest Hospital, East Sussex Healthcare NHS Trust, St. Leonards-on-Sea, GBR; 4 Respiratory Medicine, Conquest Hospital, East Sussex Healthcare NHS Trust, St. Leonards-on-Sea, GBR

**Keywords:** mri cardiac, covid-19 myositis, covid-19 pneumonitis, covid-19 myopericarditis, covid 19

## Abstract

A 63-year-old male, with no significant past history and not on any regular medications previously, had mild respiratory symptoms post the first dose of the AstraZeneca (Cambridge, England) coronavirus disease 2019 (COVID-19) vaccine, which were self-limiting. Following the second dose of the vaccine, he arrived at the emergency department (ED) with worsening shortness of breath.

During this admission, he was assumed to have interstitial lung disease due to a possible past history of occupational exposure. He responded to a short-term course of corticosteroids and antibiotics and was discharged home.

However, he reported again to the emergency department three weeks later, with persistent dyspnoea along with myalgia. His blood tests and imaging from scans suggested myositis, pneumonitis, and myopericarditis. Since he recently had the COVID-19 AstraZeneca vaccine, it was postulated as the most likely cause of the symptoms. He was managed with intravenous (IV) corticosteroids followed by oral corticosteroids with symptom resolution.

## Introduction

When dealing with the global pandemic of coronavirus disease 2019 (COVID-19), there was an urgent need for immunity that could boost the natural immune system. The answer was herd immunity from the various vaccines. on the market, recommended by the World Health Organization (WHO) [1]. The WHO issued emergency use listings (EULs) for two versions of the AstraZeneca/Oxford (Cambridge, England) COVID-19 vaccine [2]. There was funding, including indemnity given by governments across the globe to accelerate the mass production and availability of COVID-19 vaccines to the masses [3]. Vaccines and, in particular, adjuvant contents of vaccines have been shown to increase the antigen-specific immune response toward immunogenicity. This may also have negative outcomes, inducing autoimmunity [4].

We present a unique case with combined pneumonitis, myositis and myopericarditis post-COVID-19 vaccination, which responded to conservative management, highlighting the need to be aware of the prompt management of such patients with favourable outcomes.

## Case presentation

A 62-year-old male had the first dose of the AstraZeneca/Oxford COVID-19 (ChAdOx1 S {recombinant}) vaccine (AZ) three months after recovery from asymptomatic COVID-19 infection, He developed mild respiratory symptoms and was managed in primary care with oral antibiotics. Respiratory tract symptoms recurred after the second dose of the AZ vaccine two months later; he was again managed in primary care with a course of antibiotics for a lower respiratory tract infection.

**Figure 1:**
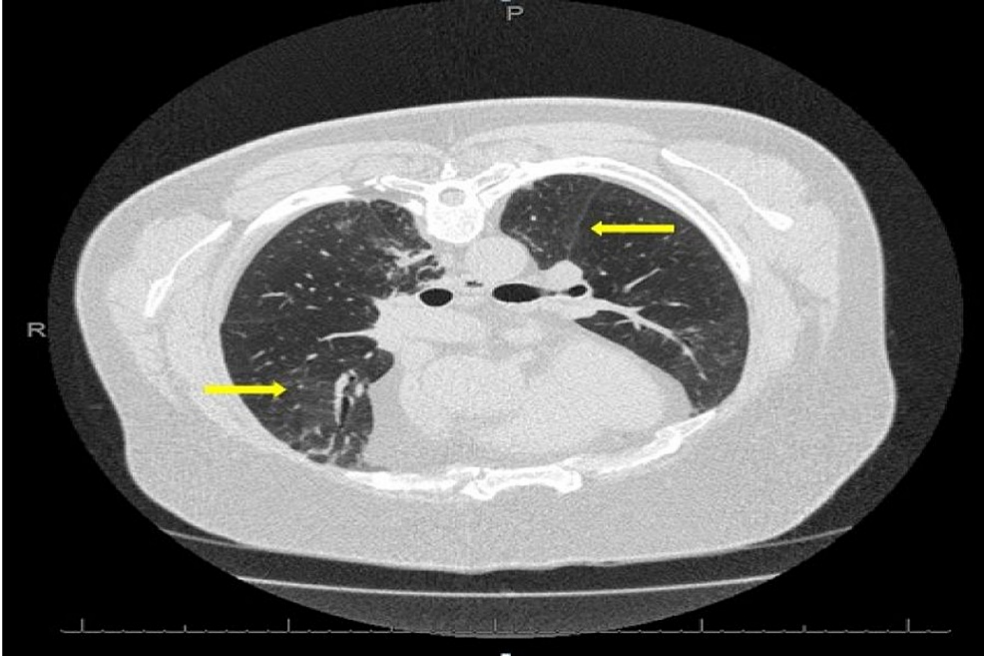
HRCT-axial view There is diffuse and patchy ground-glass attenuation (yellow arrows) with tiny nodules in the upper and mid zones, and there are multifocal areas of peripheral consolidation with tractional bronchiolar dilatation within both lower lobes. HRCT: high-resolution computed tomogram

After three weeks with persisting symptoms of dyspnoea and chest tightness, he arrived at the emergency department and was referred to the medical team on-call for further assessment. His initial observations showed oxygen saturation of 88% on air and improvement to 94% with 2 litres of oxygen. As a landlord, there was a possible exposure to bird droppings and dust at building sites while visiting various properties he was renovating. Initial blood tests and high-resolution computed tomogram (HRCT) suggested an impression of probable hypersensitivity pneumonitis (Figure 1). He was started on a weaning course of steroids with outpatient follow-up in the respiratory clinic.

Twelve days later, he presented again to the emergency department with a referral to the medical team for review. This time, he was admitted to the respiratory ward in insolation with significant myalgia, weakness and dyspnoea and was detected to have very high creatinine kinase (CK) levels of 4053 u/L (22-198), troponin levels of 472 (0-< 14) ng/ml, aspartate transaminase (AST) of 217 u/L (1-45) and C-reactive protein of 60 (-5) (Table 1).

**Table 1:** Progressive downward trend of biochemical blood markers towards resolution

Parameter	Day 1	Day 2	Day 3
Troponin	472	399	204
Creatinine Kinase (CK)	4053	-	1526
Aspartate Transaminase (AST)	217	-	88
C-Reactive Protein (CRP)	60	27	6

**Figure 2:**
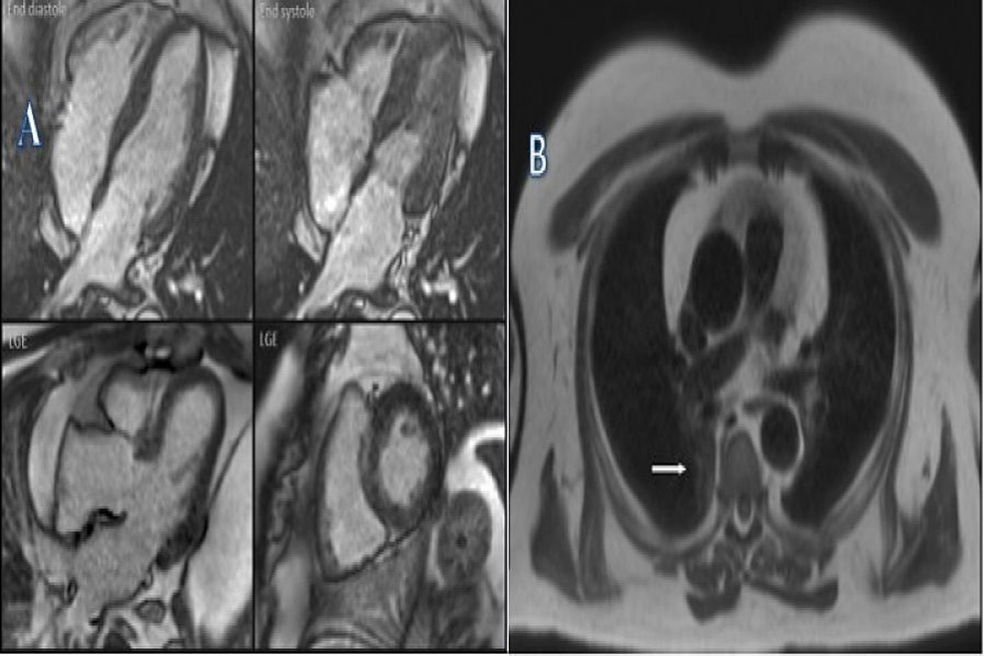
Cardiac MRI A. STIR-T2 images: Mild fibrosis of the basal septum and inferior and lateral walls. No myocardial inflammation or infarction. B. Gadolinium study: In the late phase, there is a mild mid-wall enhancement in the basal septum and inferior and lateral walls. There is an area of increased signal in the posterior right lung (white arrow). STIR: short tau inversion recovery

**Figure 3:**
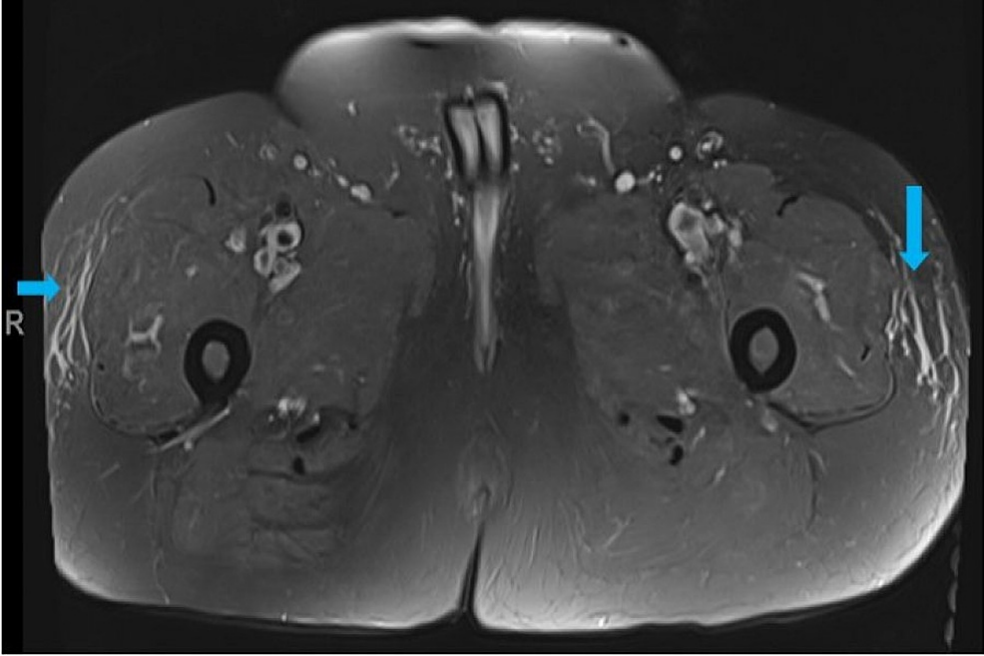
MRI-left quadriceps: axial view There is diffuse oedema in the subcutaneous fat tissue on the anterolateral sides of both thighs (blue arrows).

Although the ECG was non-specific, cardiac magnetic resonance imaging (MRI) did show mild fibrosis of the septum and inferior and lateral walls (Figure 2). MRI of the left quadriceps showed non-specific sub-cutaneous fat tissue oedema (Figure 3).

**Figure 4:**
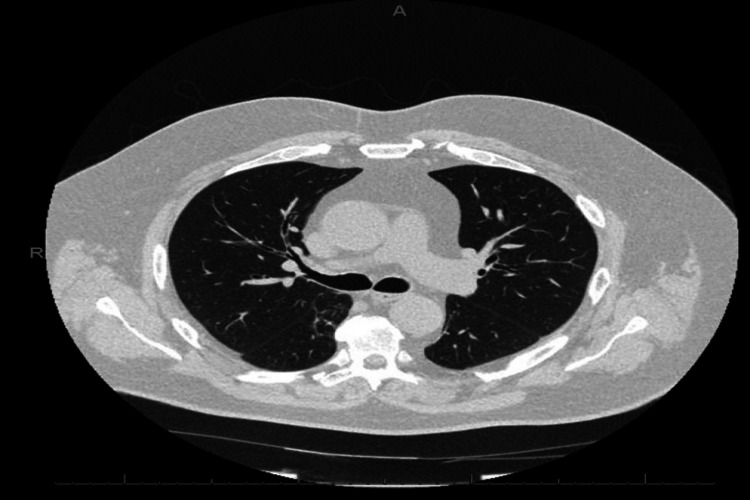
HRCT-repeat: axial view Almost complete resolution of peripheral areas of radiologically presumed organising pneumonia. HRCT: high-resolution computed tomogram

A clinical diagnosis of probable AZ COVID-19 vaccine-related inflammatory myositis, myopericarditis and pneumonitis was made. He was managed with IV methylprednisolone for three days followed by oral prednisolone 40 mg daily with weekly tapering, with appropriate proton pump inhibitor (for acid protection) and vitamin D (bone protection) while on steroids. The anti-nuclear antibody (ANA) level was 1:80 while extra-nuclear antibody (ENA) and double-stranded deoxyribonucleic antibody (dsDNA) were negative. There was a gradual improvement of CK, troponin, AST and CRP levels noted. His repeat HRCT also showed significant resolution (Figure 4). Following recovery, the patient was discharged and booked for outpatient follow-up by the rheumatology and respiratory teams.

## Discussion

A number of studies have suggested an association of co-morbidities with the COVID-19 infection [5-8]. Similarly, a number of side effects have also been reported with the use of the vaccines. Some were immediate while others present in a sub-acute fashion. These include COVID-19 vaccine-related rashes [9] and dural venous sinus thrombosis [10]. Side effects related to pneumonitis, which has been demonstrated in transgenic mice studies [11]. A number of studies have demonstrated post-COVID-19 vaccine-related myositis [12-14]. There was also cardiovascular involvement including myocarditis and myopericarditis [15-16].

It is questionable whether the introduction of a booster vaccination would have any further impact on side effects [17-18]. Additionally, National Institute for Clinical Excellence (NICE, UK) and Centre for Disease Control (CDC, USA) guidelines would need to be updated. Fortunately, the monoclonal antibody treatment by AstraZeneca has been approved by the Food and Drug Administration (FDA) USA for emergency use on December 8, 2021, for the long-term prevention of COVID-19 among people with weakened immune systems before they have been exposed to coronavirus with six-monthly injections [19]. The UK's Medicines and Healthcare products Regulatory Agency (MHRA) made the announcement on December 31, 2021, for the pill - Paxlovid, towards reducing the risk of hospitalisation and death [20]. Hopefully, these steps would ensure more safety and more favourable outcomes with a lower incidence of side effects.

We present here a unique case report of a patient who developed an augmented reaction to the second dose of the COVID-19 vaccine with pneumonitis, myopericarditis and myositis that responded well to the tapering doses of steroids. While it would be interesting to see other cases that may be reported and the outcomes of their management, it gives us some hope that there is treatment available to manage such outcomes, albeit short term.

## Conclusions

Symptomatic management with corticosteroids, either intravenous or oral, depending on the patient’s status is advised and has shown good results. Since, irrespective of vaccination, the incidence of various side effects, including myositis, pneumonitis, myopericarditis and widespread rashes, is more frequent, there is a need to update NICE UK and CDC USA guidelines and for more awareness of management.

In particular, there is an increasing need to update the guidelines related to side effects with plans to roll out booster vaccinations in the upcoming autumn season.
